# Hybrid Closed-Loop System Achieves Optimal Perioperative Glycemia in a Boy With Type 1 Diabetes: A Case Report

**DOI:** 10.3389/fped.2021.625390

**Published:** 2021-04-29

**Authors:** Jesus Dominguez-Riscart, Nuria Buero-Fernandez, Ana Garcia-Zarzuela, Fernando A. Marmolejo-Franco, Ana C. Perez-Guerrero, Alfonso M. Lechuga-Sancho

**Affiliations:** ^1^Servicio de Pediatría, Hospital Universitario Puerta del Mar, Cádiz, Spain; ^2^Instituto de Investigación e Innovación Biomédica de Cádiz (INiBICA), Hospital Universitario Puerta del Mar, Universidad de Cádiz, Cádiz, Spain; ^3^Servicio de Cirugía Pediátrica, Hospital Universitario Puerta del Mar, Cádiz, Spain; ^4^Servicio de Anestesiología y Reanimación, Hospital Universitario Puerta del Mar, Cádiz, Spain; ^5^Departamento Materno Infantil y Radiología, Facultad de Medicina, Universidad de Cádiz, Cádiz, Spain

**Keywords:** artificial pancreas, case report, children, diabetes, glucose control, hybrid closed loop system, surgery

## Abstract

The goal in type 1 diabetes (T1D) therapy is to maintain optimal glycemic control under any circumstance. Diabetes technology is in continuous development to achieve this goal. The most advanced Food and Drug Administration- and European Medicines Agency-approved devices are hybrid closed-loop (HCL) systems, which deliver insulin subcutaneously in response to glucose levels according to an automated algorithm. T1D is frequently encountered in the perioperative period. The latest international guidelines for the management of children with diabetes undergoing surgery include specific adjustments to the patient's insulin therapy, hourly blood glucose monitoring, and intravenous (IV) insulin infusion. However, these guidelines were published while the HCL systems were still marginal. We present a case of a 9-year-old boy with long-standing T1D, under HCL system therapy for the last 9 months, and needing surgery for an appendectomy. We agreed with the family, the surgical team, and the anesthesiologists to continue HCL insulin infusion, without further adjustments, hourly blood glucose checks or IV insulin, while monitoring closely. The HCL system was able to keep glycemia within range for the total duration of the overnight fast, the surgery, and the initial recovery, without any external intervention or correction bolus. This is, to the best of our knowledge, the first reported pediatric case to undergo major surgery using a HCL system, and the results were absolutely satisfactory for the patient, his family, and the medical team. We believe that technology is ripe enough to advocate for a “take your pump to surgery” message, minimizing the impact and our interventions. The medical team may discuss this possibility with the family and patients.

## Introduction

As diabetes technologies evolve toward the artificial pancreas pursuing to achieve the maintenance of the patient's glycemia within normal range, the number of children with type 1 diabetes (T1D) using these technologies is increasing. Since T1D is a relatively common condition, we often find children with T1D presenting for procedures or surgery requiring fasting and anesthesia (1). Indeed the International Society for Pediatric and Adolescent Diabetes published the clinical practice consensus guidelines for the management of children and adolescents with diabetes requiring surgery in 2018, which have been endorsed in 2019 by the recommendations proposed by a large tertiary care with a broad experience in managing such patients in a surgical setting (1, 2). The general recommendation for surgeries in which anesthesia is predicted to last for more than 2 h or is likely to cause post-operative nausea, inability to feed, or vomiting include receiving both dextrose and insulin *via* intravenous (IV) infusion, hourly blood glucose (BG) checks, and specific adjustments in the patient's insulin schedule. These recommendations, however, focus on patients using continuous subcutaneous insulin infusion (CSII) with predicted low glucose suspend algorithm (also known as sensor-augmented CSII), but not to the most advanced devices.

Technologies evolve at a faster pace than scientific recommendations for every aspect of diabetes care. The latest European Medicines Agency- and Food and Drug Administration-approved insulin pumps are the hybrid closed-loop (HCL) systems, a leap toward the artificial pancreas, which are already being used by an increasing number of patients, both with T1D and T2D. The HCL systems basically administer subcutaneous insulin in the form of micro-boluses according to an automated algorithm in response to glucose levels as measured by a glucose continuous monitor (3). The HCL systems are programmed by the physician initially to a certain insulin administration schedule. During the first weeks of use, this system functions as a sensor-augmented CSII and continuously collects information on the patients' response to the preprogrammed insulin. It then applies artificial intelligence algorithms to adapt to each individual's response. After a period of deep learning (minimum of 2 weeks), the pump may be set to work in “auto-mode,” that is, responding automatically to glucose levels without a fixed basal rate. As a back-up emergency plan, in the event of temporary CGM signal interruption or loss, the HCL system keeps running according to the initial program (manual mode) as a conventional CSII. When in auto-mode, the HCL systems have proven to obtain enhanced glycemic control and reduced variability (improved time in glycemic target range), fewer hypoglycemic episodes, and greater patient satisfaction (4, 5).

We present a case of a boy with long-standing T1D, using HCL system therapy for months with good results, who needed surgery for an appendectomy. Since there are still no recommendations for the use of these devices in the perioperative period, we agreed with the patient, the family, and the surgical and anesthesiology team to continue with the HCL system throughout the whole procedure under the strict supervision of pediatric endocrinologists.

## Patient Presentation

A 9-year-and-9-month-old boy with uncomplicated autoimmune T1D since the age of 11 months, 46.3 kg of weight, and with HbA1c of 7% in his last consultation 2 months before the event, was admitted to the emergency room with abdominal pain migrating to the right lower quadrant, anorexia, and nausea during the last 24–48 h. The blood tests showed 8,620/mm^3^ white blood cell count with 60% neutrophils and 10.5 mg/L C-reactive protein. There were no signs of diabetic ketoacidosis, and the glucose levels were optimal, with negative B-hydroxybutyrate [pediatric appendicitis score (6) = 7]. Abdominal ultrasounds confirmed an increased appendix thickness (8 mm) and diagnosis of uncomplicated appendicitis.

**Figure 1:**
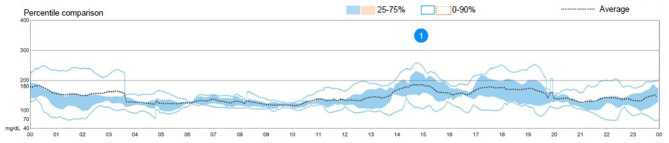
Graph showing an overview of the patient's glycemic control the week prior to the episode. Directly taken from the patient's personal record on the hybrid closed-loop system's manufacturer's site.

The patient had used a HCL system (Medtronic 670G® in auto-mode) for 9 months, had improved metabolic control since its use (estimated HbA1c 7.3% and average blood glucose 162 ± 68 mg/dl; Figure 1), felt very comfortable with the device, and both the patient and the parents were reluctant to interrupt it (the system's program details are shown in Supplementary Table 1). His ratios and sensitivities had been programmed in his last visit according to the patient's glycemic response to prandial insulin, but this does not affect basal insulin administration since the basal rates are not altered by this program when the system runs in auto-mode. According to data extracted from the patient's account on the web managing system of the pump, at 2 days prior to the symptoms, the system's basal delivery had been 20.6 and 19.2 IU/day, respectively. Since the first symptoms presented, a remarkable increase in these needs were noted, with a basal delivery rate of 26.7 and 30.7 IU/day, respectively.

Upon diagnosis, the patient was started on intravenous cefoxitin at 40 mg/kg and acetaminophen. We then faced an overnight fast since surgery was programmed for the next morning. The parents expressed their desire to continue with the HCL system if it were possible throughout the procedure. Since there are still no recommendations for this devices, we first checked that the CGM had still 72 h left of expected life and that the pump had enough insulin for at least 36 more hours. The CSII was inserted at the left gluteus and the CGM on the left arm; thus, neither would interfere with the surgical field or with the electric scalpel.

**Figure 2:**
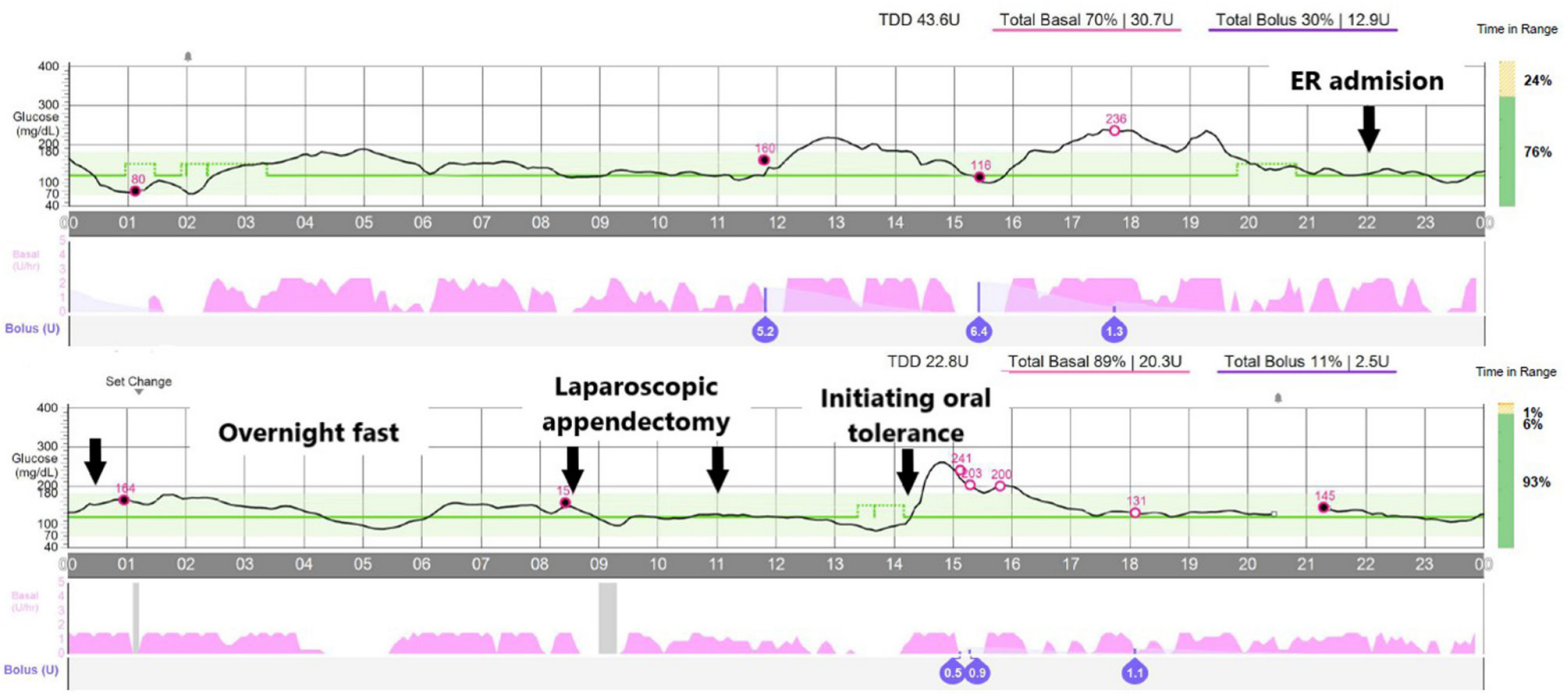
Overview of the glucose levels along the episode. Arrows point to the following events: (1) admission at the emergency room, (2) overnight fast, (3 and 4) laparoscopic appendectomy, and (5) initiating oral tolerance. Open circles reflect blood glucose calibrations.

Since all prerequisites were fulfilled and the family gave written consent, the pediatric endocrinology team agreed to let the system run in auto-mode, under close clinical monitoring. The pump's basal rate in case the system switched to “manual mode” due to CGM loss or malfunction was adjusted to 50% of the usual dose as recommended (7). We decided to start an IV infusion of 5% dextrose, with 150 mEq/L of sodium and 20 mEq/L of potassium to prevent ketosis (see Table 1 for preparation instructions), but avoided IV insulin and checked the system's behavior overnight. Indeed capillary ketones were always under 0.3 (we checked every 4 h), and glucose levels were always in the 80-180 mg/dl (4.44–9.99 mmol/L) range. The system infused a total of 82 micro-boluses of 0.11 IU of insulin on average (range, 0.03–0.13; Figure 2—overnight fast). The system also spent a total time of 1 h and 15 min without administering any micro-bolus.

**Table 1:** Summary of recommendations on the use of the hybrid closed-loop system during the perioperative period.

**Pre-surgery**	- Check if the pump battery is charged - Check if the CGM has at least 72 h of expected lifetime and the sensor is properly calibrated - Check if there is enough insulin at the reservoir for at least 36 h - Check if the transmitter is successfully communicating with the pump - Check if the location of neither the sensor nor the catheter may interfere with the surgery field or the electric scalpel - Start infusion of 5% IV dextrose, with 150 mEq/L sodium and 20 mEq/L potassium (prepare by adding 22 ml of 20% NaCl and 5 ml of 2 M KCl to every 500 ml of 5% dextrose) - Check capillary ketones every 4 h - Check if the suspension before low and high management settings is “ON” - Check if the high, low, and before alerts are “ON” - Audio mode is “ON” - Airplane mode is “OFF” - Check if the insulin bolus is not being delivered. - Check if the glucose level is within optimal range.
**During surgery**	- Follow the indications on the pump - Should glucose levels rise over 200 mg/dl or fall below 70 mg/dl on the systems screen, check the capillary blood glucose levels with a glucose strip. - Stop pump infusion and start IV insulin perfusion if the glucose levels rise over 250 mg/dl (after checking with capillary blood glucose) despite pump correction bolus - Consider changing the dextrose concentration in perfusion to 10% or administering a bolus of 10% dextrose IV (10 ml/kg) if the glucose level falls under 70 mg/dl, as checked in a capillary or venous sample

*CGM, continuous glucose monitor; IV, intravenous*.

Having analyzed the system's performance, we agreed with the anesthesiologist and the surgeons to keep the system running automatically throughout the procedure, having a clear plan for CSII interruption and insulin IV infusion if necessary. A member of the pediatric endocrinology team was available at all times.

The laparoscopic appendectomy took ~2.5 h since the patient was sent to the surgery room until he left it, and it was performed without relevant incidences. Pathologic confirmation of acute uncomplicated appendicitis was obtained. The system automatically delivered 16 micro-boluses during the procedure. The mean dose of these boluses was 0.089 IU of insulin (range, 0.05–0.13), and the glucose levels were always within range (Figure 2—laparoscopic appendectomy). Taking together the overnight fast and the surgery, the system infused 20.3 IU/day of insulin, in contrast with the 30.7 IU/day it had infused the day before.

Right before oral tolerance was started, the patient's mother noticed that the glucose levels tended to drop and programmed, by her own initiative, a temporary increase in the glucose target to 140 mg/dl. We then decided to begin oral tolerance (3 h post-surgery; Figure 2), resulting in a hyperglycemic peak (maximum, 280 mg/dl) since no prandial insulin bolus had been administered, and dextrose fluid had not been discontinued.

The patient was finally discharged 24 h after surgery without incidence and with the HCL system functioning uninterruptedly. The basal insulin infusion at 48 h after surgery dropped down to 19.0 and 15.5 IU/day.

## Discussion

To the best of our knowledge, we present the first pediatric case of a patient with T1D undergoing a major surgery with uninterrupted use of an HCL system running in auto-mode. Our results were optimal in terms of glycemic control, with no external or additional adjustments, as well as the patient's and family's satisfaction. This approach allowed for a minimally invasive and less disruptive management, performed in agreement with the family and surgical team and with the pediatric endocrinologist's commitment to closely supervise the procedure throughout. This was necessary since the most recent clinical guidelines and recommendations for the management of patients with diabetes in CSII therapy undergoing surgery or anesthesia-requiring procedures focus on conventional CSII and do not take into account the more advanced HCL systems (1, 2). A previous report on an adult patient with T1D undergoing metabolic surgery (sleeve gastrectomy) focused more on the results of the surgery itself rather than the perioperative period but helped us in the decision to program the 50% reduction in the manual-mode basal insulin delivery rates (7).

Insulin doses administered as micro-boluses automatically by the pump in response to the patient's CGM were 0.024 IU/kg/h during fasting and 0.015 UI/kg/h during appendectomy. To achieve such low-dose infusion with IV insulin, we would have very likely needed to check the patient's BG hourly and make adjustments to the insulin and dextrose infusions, which is how it is recommended to date.

Alternatively, our approach specifically allowed us to avoid interrupting the patient's usual therapy, hourly capillary BG testing, the use of alternative basal rate, and the need for intravenous insulin infusion. Continuing the patient's usual therapy was less disruptive and invasive and resulted in the patient's, family's, and medical team's satisfaction.

Since diabetes technology advances at an accelerated pace, it seems very unlikely that we have time to develop well-dimensioned clinical trials to test every technical step forward in every aspect of diabetes care, especially how to face unexpected events such as urgent surgeries. We believe that this experience provides a starting point for a novel approach to perioperative management of children with T1D and advocate for a “take your pump to surgery” message that could be contemplated with patients and family and considered by surgical teams, under close supervision by pediatric endocrinologists. We provide a table of recommendations based on previous recommendations (1, 2), reports (7), and our own experience (Table 1).

## Data Availability Statement

The original contributions presented in the study are included in the article/Supplementary Material, further inquiries can be directed to the corresponding author/s.

## Ethics Statement

Ethical review and approval was not required for the study on human participants in accordance with the local legislation and institutional requirements. Written informed consent to participate in this study was provided by the participants' legal guardian/next of kin. Written informed consent was obtained from the minor(s)' legal guardian/next of kin for the publication of any potentially identifiable images or data included in this article.

## Author Contributions

JD-R and NB-F contributed to the clinical management of the case, drafted the initial manuscript, and reviewed and revised the manuscript. AG-Z acquired data, performed the analyses and interpretation of data, and critically revised the manuscript for important intellectual content. FM-F and AP-G agreed to perform the surgery and anesthesia, letting the HCL system take care of the glycemic control, thus contributing to the conception and design of the study, and reviewed and revised the manuscript. AL-S conceptualized and designed the clinical management strategy, coordinated and supervised the procedure and data collection, and wrote the final version of the manuscript. All the authors approved the final manuscript as submitted and agree to be accountable for all aspects of the work.

## Conflict of Interest

The authors declare that the research was conducted in the absence of any commercial or financial relationships that could be construed as a potential conflict of interest.
