# Supplementary material for: Hybrid Closed-Loop System Achieves Optimal Perioperative Glycemia in a Boy With Type 1 Diabetes: A Case Report
Source: Front Pediatr. 2021 Apr 29;9:625390. doi: 10.3389/fped.2021.625390 (PMC8116592; doi:10.3389/fped.2021.625390)
Supplement: Supplementary file 1 [file Table_1.DOCX]

Table 1. The system’s manual mode program at presentation:

| Time period | CHO Ratio (g/IU) | Sensitivity | Glucose target (mg/dl) |
| --- | --- | --- | --- |
| 00:00 – 07:00 | 33 | 95 | 125 |
| 07:00 – 11:00 | 6.1 | 87 | 105 |
| 11:00 – 13:00 | 6.7 | 72 | 105 |
| 13:00 – 17:00 | 7 | 70 | 105 |
| 17:00 – 21:00 | 5.2 | 53 | 105 |
| 21:00 – 24:00 | 6.8 | 65 | 110 |
